# Humanized HLA-DR4.RagKO.IL2RγcKO.NOD (DRAG) mice sustain the complex vertebrate life cycle of *Plasmodium falciparum* malaria

**DOI:** 10.1186/1475-2875-13-386

**Published:** 2014-09-30

**Authors:** Wathsala Wijayalath, Sai Majji, Eileen F Villasante, Teodor D Brumeanu, Thomas L Richie, Sofia Casares

**Affiliations:** US Military Malaria Vaccine Program, Naval Medical Research Center/Walter Reed Army Institute of Research, 503 Robert Grant Avenue, Silver Spring, MD 20910 USA; Department of Medicine, Uniformed Services University of Health Sciences, 4301 Jones Bridge Road, Bethesda, MD 20814 USA; Sanaria Inc, 9800 Medical Center Drive, Rockville, MD 20850 USA

**Keywords:** Malaria, *Plasmodium falciparum*, Human-immune-system humanized mice, Hepatocytes, Kupffer cells, Liver endothelial cells, Erythrocytes, Antibodies, Cellular-mediated immunity

## Abstract

**Background:**

Malaria is a deadly infectious disease affecting millions of people in tropical and sub-tropical countries. Among the five species of *Plasmodium* parasites that infect humans, *Plasmodium falciparum* accounts for the highest morbidity and mortality associated with malaria. Since humans are the only natural hosts for *P. falciparum*, the lack of convenient animal models has hindered the understanding of disease pathogenesis and prompted the need of testing anti-malarial drugs and vaccines directly in human trials. Humanized mice hosting human cells represent new pre-clinical models for infectious diseases that affect only humans. In this study, the ability of human-immune-system humanized HLA-DR4.RagKO.IL2RγcKO.NOD (DRAG) mice to sustain infection with *P. falciparum* was explored.

**Methods:**

Four week-old DRAG mice were infused with HLA-matched human haematopoietic stem cells (HSC) and examined for reconstitution of human liver cells and erythrocytes. Upon challenge with infectious *P. falciparum* sporozoites (NF54 strain) humanized DRAG mice were examined for liver stage infection, blood stage infection, and transmission to *Anopheles stephensi* mosquitoes.

**Results:**

Humanized DRAG mice reconstituted human hepatocytes, Kupffer cells, liver endothelial cells, and erythrocytes. Upon intravenous challenge with *P. falciparum* sporozoites, DRAG mice sustained liver to blood stage infection (average 3–5 parasites/microlitre blood) and allowed transmission to *An. stephensi* mosquitoes. Infected DRAG mice elicited antibody and cellular responses to the blood stage parasites and self-cured the infection by day 45 post-challenge.

**Conclusions:**

DRAG mice represent the first human-immune-system humanized mouse model that sustains the complex vertebrate life cycle of *P. falciparum* without the need of exogenous injection of human hepatocytes/erythrocytes or *P. falciparum* parasite adaptation. The ability of DRAG mice to elicit specific human immune responses to *P. falciparum* parasites may help deciphering immune correlates of protection and to identify protective malaria antigens.

## Background

Malaria infection is initiated by the bite of an infected female *Anopheles* mosquito and inoculation of sporozoites in the skin that rapidly migrate through the bloodstream to infect hepatocytes. Mature liver stage parasites are then released to the bloodstream to invade red blood cells (RBCs) and to initiate the asexual erythrocytic cycles responsible for the clinical manifestations of malaria [1]. Among the five species of *Plasmodium* that infect humans, *Plasmodium falciparum* is the most virulent with 1.2 billion people at high risk. The number of *P. falciparum* deaths reported by WHO for 2012 was 627,000, while the Institute of Health Metrics and Evaluation reported 1.24 million deaths for 2010 [2], the same year when WHO reported 655,000 deaths. Malaria is a disease of poverty and a cause of poverty as it has deeply slowed economic growth in malaria endemic areas. The current rise of earth temperature could hasten mosquito breeding at higher altitudes and latitudes and might increase the burden of malaria across the globe [3].

Since humans are the only species that can be naturally infected with *P. falciparum,* the development of efficacious malaria vaccines and anti-malarial drugs has been hindered by the lack of convenient animal models. The ability of great apes (chimpanzees, gorillas and bonobos) to sustain *P. falciparum* natural infection is controversial, though they develop malaria upon experimental challenge with sporozoites [4, 5]. The use of great apes for biomedical research is currently under moratorium [6]. New World monkeys are susceptible to experimental infection with *P. falciparum* blood stage parasites that have been previously adapted to grow in monkeys, and splenectomy is vital for maintenance of long-term infections [7]. Except for one *Aotus* strain (*Aotus lemurinus griseimembra*), New World monkeys do not sustain reproducible *P. falciparum* liver stage infection [7], which further poses a challenge for testing pre-erythrocytic drugs and vaccines. Studies in rodent models of malaria have significantly advanced our understanding of the parasite biology but it has become clear that rodent malaria does not represent the complexity of *P. falciparum* and that vaccines that were able to protect mice against murine parasites did not protect humans against *P. falciparum* when tested in clinical trials. There are relevant differences between rodent malaria parasites and *P. falciparum.* The duration of liver stage infection is two days for rodent malaria parasites, but 5–7 days for *P. falciparum* [8]. Some antigenic proteins expressed by *P. falciparum-*infected hepatocytes or infected RBCs do not have orthologs for rodent malaria parasites, as is the case of liver stage antigen 1 (LSA1) [9] and the polymorphic or “variant” proteins encoded by *var*, *rif*, *stevor*, and *Pfmc-2TM,* which are responsible for immune evasion [1]. Some proteins shared by *P. falciparum* and rodent malaria parasites do not exert the same biological function [10]. Though *P. falciparum* sporozoites do not invade and develop in mouse hepatocytes [11, 12], mice are often used to assess the immunogenicity of *P. falciparum* pre-erythrocytic sub-unit vaccine candidates, particularly for the circumsporozoite protein (CSP), which is highly expressed on sporozoites. This approach is however challenged by the differential ability of mouse Major Histocompatibility Complex (MHC) *vs* human MHC (HLA) molecules to present malaria T cell epitopes, which may drive different cellular and humoral responses to *P. falciparum* antigens [13].

To overcome these limitations much effort has been devoted to generate humanized mouse models for *P. falciparum*. Immunodeficient mice transgenic for urokinase-type plasminogen activator transgene (*uPa,* SCID/ *uPa* mice) or knockout for fumarylacetoacetate hydrolase (Fah, FRG mice) transplanted with human hepatocytes have proven to sustain *P. falciparum* liver stage infection [11, 14, 15] and to transition toward blood stage infection upon exogenous injection of human erythrocytes [11]. Likewise, immunodeficient mice injected daily with very large numbers of human RBCs can sustain *P. falciparum* blood stage infection upon challenge with *P. falciparum* mouse-adapted blood stage parasites but only for a short period of time, since the human RBCs rapidly disappear from blood circulation upon cessation of RBC daily injections [16–20] *.* The lack of a human-immune-system in these models also prevents investigating human immune responses to *P. falciparum*.

Human-immune-system (HIS) humanized mice represent new pre-clinical models for human infectious diseases and for testing human vaccines. Studies demonstrated that expression of HLA class II molecules on HIS mice favors human haematopoietic stem cell (HSC) engraftment, human T cell development, and human B cell immunoglobulin class switching [21, 22]. One of these models, namely DRAG (HLA-DR4.RagKO.IL2RγcKO.NOD) mice, when infused with HLA II-matched human HSC, develop functional human T and B cells, reconstitute serum levels of all human immunoglobulin classes and subclasses (natural antibodies), and respond to vaccination by eliciting specific antibodies [21]. This study demonstrates that humanized DRAG mice also reconstitute human hepatocytes, Kupffer cells, liver endothelial cells, and erythrocytes/reticulocytes, and sustain the full vertebrate life cycle of *P. falciparum*. Infected DRAG mice elicited specific antibody and cellular responses and self-cured the infection by day 45 after the challenge.

## Methods

### Mice

Animal procedures reported herein were conducted under protocols approved by the Institutional Animal Care and Use Committees at the Walter Reed Army Institute of Research/Naval Medical Research Center and at the Uniformed Services University of Health Sciences in compliance with the Animal Welfare Act and in accordance with the principles set forth in the “Guide for the Care and Use of Laboratory Animals,” Institute of Laboratory Animals Resources, National Research Council, National Academy Press, 1996. The HLA-DR4.Rag1KO.IL2RγcKO.NOD (DRAG) mice were bred at NMRC/WRAIR Veterinary Service Program. Procedures for infusion of HLA-DRB1*0401-positive human HSC from umbilical cord blood have been previously described [21]. DRAG mice were used at four months post-infusion of human HSC.

### Immunohistochemistry

Livers were separated into lobes, embedded in Tissue-tek O.C.T. compound (Sakura Finetek, Torrance, CA) and immediately frozen at −80°C. Serial 8-μm cryo-sections were stored at −80°C until use. Slides were thawed for 30 min at room temperature and fixed either with cold absolute methanol for 10 min followed by cold acetone for 1 min (for hepatocyte and liver-stage parasite immunostaining) or with BD Cytofix/Cytoperm fixation/permeabilization kit (for Kupffer cell and endothelial cell immunostaining). Following fixation, sections were washed three times with PBS, blocked with PBS/1% BSA for 30 min at 37°C, washed again and incubated with primary antibodies for 30 min at 37°C, washed again and incubated with secondary antibodies for 30 min at 37°C. The primary antibodies used were mouse anti-human hepatocyte specific antigen (Hep Par-1, clone #OCH1E5, Santa Cruz Biotechnology, Dallas, TX), mouse anti-human CD68 (clone #Y1/82A, BDbiosciences,), mouse anti-human CD36 (clone #CB38, BDbiosciences,) or rabbit anti-*P. falciparum* heat shock protein 70 (PfHSP70) antibody (LifeSpan Biosciences, Seattle, WA). Secondary antibodies were FITC-goat anti-rabbit IgG (H + L) (KPL, Gaithersburg, MD), Rhodamine goat anti-mouse IgG (H + L) (KPL), FITC-goat anti-mouse IgG2b (Southern Biotech, Birmingham, AL), and FITC-goat anti-mouse IgM (Southern Biotech). Slides were washed, air dried, and mounted in Vectashield mounting medium with DAPI (Vector Laboratories, Burlingame, CA). Thin blood smears were fixed with 0.05% glutaraldehyde and permeabilized with 0.1% Triton-X100, blocked with PBS/1% BSA for 30 min at RT, and stained with mouse anti-human haemoglobin A (Ray Biotech, Norcross, GA) followed by anti-mouse IgG1-FITC (Southern Biotech). Slides were examined under fluorescence microscopy (wide-field for hepatocytes, Kupffer cells and endothelial cells or confocal for liver stage malaria parasites). Wide-field fluorescence images were acquired using QCapture Pro 6.0 software in Olympus BX51 microscope and confocal images were acquired using LaserSharp2000 software in Zeiss/Bio Rad Laser Confocal Microscope. For human transferrin staining, sections from 10% formalin fixed, paraffin-embedded livers were subjected to antigen-retrieval, stained with anti-human transferrin (Histoserv, Germantown, MD), and examined under light microscopy.

### Isolation of spleen cells, bone marrow cells, hepatocytes and Kupffer cells

Splenic and bone marrow cells were isolated as described [21]. Livers were perfused as described [23] using liver perfusion medium (Life Technologies, Grand Island, NY) followed by liver digest medium (0.8 mg/mL collagenase type I in DMEM). Liver cells were suspended in wash solution (2% BSA/10 mM Hepes/2 mM EDTA/2 mM β mercaptoethanol/DMEM), passed through 100-micron cell strainers and washed three times at 50 g for 2 min to pellet hepatocytes. Following each wash, the supernatants were collected, spun at 1350 g for 10 min, cell pellets were combined and washed thrice with wash solution to collect the cell fraction containing Kupffer cells.

### FACS analysis

Kupffer cells were washed with PBS/1% BSA/0.1% NA azide (staining buffer), blocked with Fc block (BDbiosciences, San Jose, CA) for 10 min on ice, incubated with anti-human CD45 (BDbiosciences) for 30 min, followed by intra-cellular staining with anti-human CD68 (clone# Ki-M7, AbD serotec, Raleigh, NC) using BD Cytofix/Cytoperm fixation/permeabilization kits (BDbiosciences). Hepatocytes were washed with staining buffer followed by intra-cellular staining with goat anti-human serum albumin (MyBiosource, San Diego, CA). Approximately 5 million events were acquired for each staining. Peripheral blood (1–2 μl) drawn from the vein of the tail was collected in heparin-coated capillaries (Sigma-Aldrich, St Louis, MO), washed twice with PBS, suspended in PBS/1% BSA, blocked with Fc Block for 15 minutes at RT and stained with anti-human CD235 (BDbiosciences) and Retic-Count (BDbiosciences) for 30 minutes. Bone marrow cells were obtained by flushing both tibias as described [21] and cell surface stained with anti-human CD45, anti-human CD71, anti-human CD235, and anti-mouse Ter-119 antibodies (BDbiosciences). Apoptosis in bone marrow cells was measured using Annexin V/7-ADD apoptosis detection kit (BD Biosciences).

### ELISA

Peripheral blood was drawn from the tail vein in heparin-coated capillaries and plasma was collected following centrifugation. Plasma levels of human transferrin were measured using anti-human transferrin ELISA kits (Bethyl Laboratories, Montgomery, TX).

### *Plasmodium falciparum*parasites

*Plasmodium falciparum* (NF54 or 3D7)-infected *Anopheles stephensi* mosquitoes were obtained from the Department of Entomology, WRAIR/NMRC. Infectious *P. falciparum* sporozoites were isolated by dissecting the salivary glands in E-199 medium (Quality Biological, Gaithersburg, MD) with 5% human AB serum (Key Biologics, Memphis, TN). Mice were challenged intravenously with *P. falciparum* sporozoites and followed for parasitaemia by PCR and thin and thick Giemsa-stained blood smears.

### Detection of malaria parasites by PCR

Mice were bled from the tail vein and DNA was extracted using DNeasy blood and tissue kits (Qiagen, Valencia, CA). A pair of *Plasmodium* genus-specific primers [24] was used to amplify all units of rRNA distributed in chromosomes 1, 5, 7, 11 and 13; Forward 5-GCTCTTTCTTGATTTCTTGGATG-3 Reverse 5-AGCAGGTTAAGATCTCGTTCG-3. PCR amplification was carried out in 20 μL reaction volume containing 50 ng of test or positive control DNA, 0.025 unit of *Taq* polymerase (Life technologies), 0.5 μM each of primers, 0.2 mM deoxynucleotide triphophates (dNTPs) (Life technologies) and 0.2 mM MgCl2. PCR settings were: 1 cycle for 95°C for 10 min followed by 41 cycles of 95°C for 30 sec, 56°C for 30 sec and 72°C for 1 min. PCR amplified products were analysed on agarose gels (3%) with ethidium bromide.

### *In vitro*culture of *P. falciparum*parasites and mosquito feeding

DRAG mice challenged with *P. falciparum* sporozoites were bled on day 14 and 18 post-challenge and 20–50 μl of blood was cultured with 5% haematocrit (O^+^ human RBCs) (Key Biologics) in RPMI 1640 supplemented with 2 mM L-glutamine (Life technologies), 25 mM Hepes, 0.36 mM hypoxanthine, 2 g/L sodium bicarbonate and 10% human AB serum (Key Biologics) at 37°C in a mixture of gas containing 5% CO_2_, 5% O_2_, and 90% N_2_. Asexual blood cultures were maintained with daily media changes and weekly subcultures in O^+^ human erythrocytes. Parasitaemia in cultures was examined by Giemsa-stained thick and thin blood smears. At 2% asexual parasitaemia, cultures were sub-cultured in fresh O^+^ human RBCs and maintained with daily media changes until the development of mature gametocytes. At 1.8-2.0% gametocytaemia, cultures were spun at 500 g for 10 min, the parasite pellet was re-suspended in 2 mL of human A^+^ serum with 0.5 mL of packed O^+^ human RBCs and starved female *An. stephensi* mosquitoes were fed on the gametocyte suspension using membrane feeders (*in vitro* feeding). For *in vivo* feeding, *An. stephensi* mosquitoes were allowed to feed on for five minutes on infected mice anaesthetized with ketamine. Unfed mosquitoes were removed and the fed mosquitoes were maintained at the Department of Entomology, WRAIR/NMRC. Midguts and salivary glands were dissected at day 7 and 14 post-feeding to determine the percentage of mosquitoes infected with oocysts and sporozoites.

### *Plasmodium falciparum*antibody titers by immunofluorescence Assay (IFA)

Teflon printed 12-well slides (Electron Microscopy Sciences, Hatfield, PA) were coated either with synchronized *P. falciparum* ring-stage parasite infected-red blood cells (iRBC) [25] or trophozoite/schizont infected RBCs [26] (6,000 RBCs/well at 6% parasitaemia, in PBS/1% BSA). Slides were air dried and stored at −80°C until use. Upon thawing, slides were blocked with PBS/1% BSA for 30 min at 37°C. Twenty microlitres of plasma at various dilutions was added to the wells and incubated for 1 h at 37°C. Slides were washed three times with PBS, incubated with FITC-labeled anti-human IgG or IgM (Southern Biotech) for 30 min at 37°C, washed, and mounted with Vectashield-DAPI (Vector Laboratories).

### T cell responses

Cultures of *P. falciparum* blood stage parasites (NF54, 10^8^, 5% parasitaemia) were pelleted and suspended in ACK (Invitrogen) for five minutes in ice to lyse the erythrocytes, washed twice in 1xPBS and the parasite pellet was freeze-thawed three times using liquid nitrogen and boiling water, followed by sonication at 75% amplitude, with 20 sec on, 10 sec off for 3 min. Cell lysates were centrifuged at 10,000 rpm/4°C for 15–20 min and protein concentration was measured by Biuret. Splenocytes (5x10^5^) were stimulated with *P. falciparum* proteins extracts (30 μg/ml) for 2 days or left unstimulated. *In vitro*-stimulated cells were incubated with Golgistop (BDbiosciences) for four hours, surface stained with human CD3, CD4 and CD8 antibodies and intracellularly stained with human TNF and IFNγ and analysed by FACS.

## Results

### DRAG mice reconstitute human hepatocytes, Kupffer cells, and liver endothelial cells

**Figure 1:**
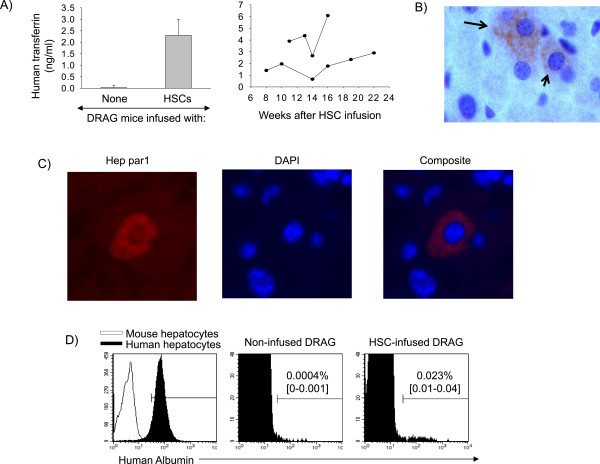
**DRAG mice develop human hepatocytes. A)** Plasma levels of human transferrin in DRAG mice (n = 14) at 14 weeks post-infusion of HSC. No human transferrin was detected in serum from control (non-HSC infused) DRAG mice (n = 7). Data represent mean ± SD of mice analysed individually **(left panel)**. Kinetics of human transferrin in plasma of two DRAG mice analysed individually **(right panel)**. **B)** Staining of liver sections with anti-human transferrin. Arrow shows the presence of human hepatocytes. **C)** Identification of human hepatocytes in livers of HSC-infused DRAG mice using human anti-Hep Par-1 (red) and counterstained with DAPI (DNA, blue). **D)** Frequency of human hepatocytes in livers of DRAG mice as measured by FACS using anti-human albumin. Left panel shows binding of anti-human albumin to primary human hepatocytes (black histogram) but not to mouse hepatocytes (white histogram). Middle and right panels show anti-human albumin staining in livers of control (non-HSC infused) and in HSC-infused DRAG mice, respectively. Data represent mean and interval frequencies from three mice analysed individually.

The ability of human HSC (CD34^+^) to differentiate into non-haematopoietic cells such as hepatocytes, cardiomyocytes, and endothelial cells has become evident in human and animal studies [27]. Thus it was investigated whether DRAG mice develop human hepatocytes by measuring plasma levels of human transferrin, a protein secreted by human hepatocytes [28]. At 14 weeks post-infusion of human HSCs the levels of human transferrin averaged 2–3 ng/ml (Figure 1A, left panel), which is about 5x10^5^ fold lower than the levels of transferrin in human plasma (1 mg/ml) [28]. No human transferrin was detected in plasma of naïve (non-HSC infused) DRAG mice. Longitudinal analysis indicated that DRAG mice reconstituted human transferrin at eight weeks post-infusion of HSCs and the levels remained constant for up to 22 weeks (Figure 1A, right panel). Histological examination using anti-human transferrin (Figure 1B) and anti-human Hep Par-1 (a marker specific for human hepatocyte mitochondria) [29] (Figure 1C) revealed the presence of scattered human hepatocytes within the livers. To estimate the frequency of human hepatocytes, livers of HSC-infused DRAG mice were analysed by FACS using anti-human albumin. As illustrated in Figure 1D, approximately 0.023% of the hepatocytes in mouse livers were of human origin. Thus the results indicated that DRAG mice infused with HSC develop human hepatocytes.

**Figure 2:**
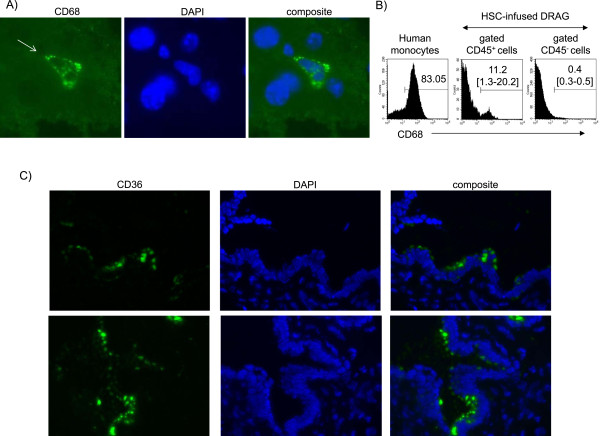
**DRAG mice develop human Kupffer cells and liver endothelial cells. A)** Immunostaining of livers using anti-human CD68 (green fluorescence) and counterstained with DAPI (DNA, blue fluorescence). **B)** Frequency of human Kupffer cells in livers of DRAG mice as determined by FACS using anti-human CD45 and anti-human CD68 on cells gated on the FSC/SSC monocyte region. Middle and right panels show CD68 staining on human (gated CD45^+^) cells and mouse (gated CD45^−^) cells isolated from livers of HSC-infused DRAG mice. Data represent mean and interval frequencies from 4 mice analysed individually. Left panel shows binding of anti-human CD68 to monocytes from human peripheral blood. **C)** Immunostaining of livers using anti-human CD36 and counterstained with DAPI (DNA, blue fluorescence) showing presence of human endothelial cells in veins and sinusoids.

Prior to infection of hepatocytes, sporozoites must cross the sinusoidal cell layer and interact with endothelial cells and Kupffer cells [30, 31]. The presence of human Kupffer cells in livers of HSC-infused DRAG was revealed by immunohistochemistry using anti-human CD68 (Figure 2A). CD68 is a glycoprotein binding to low density lipoprotein (LDL) expressed on mature (phagocytic) human Kupffer cells [32]. To further estimate the frequency of human Kupffer cells, mononuclear cells isolated from livers that had been previously perfused to remove circulating blood cells, were analysed by FACS using anti-human CD45 (a marker expressed on human haematopoietic cells) [22] and anti-human CD68. As illustrated in Figure 2B (middle panel), the frequency of Kupffer cells (CD68^+^CD45^+^) averaged 11%. The specificity of the anti-human CD68 is shown by binding to monocytes from human peripheral blood (Figure 2B left panel) but not to mouse (CD45^−^) cells from livers of HSC-infused DRAG mice (right panel). Livers from HSC-infused DRAG mice also showed the presence of human endothelial cells lining the sinusoids, as indicated by histological examination using anti-human CD36 (Figure 2C). In aggregate, the results indicated that HSC-infused DRAG mice develop human hepatocytes, Kupffer cells, and liver endothelial cells.

### DRAG mice reconstitute human erythrocytes

**Figure 3:**
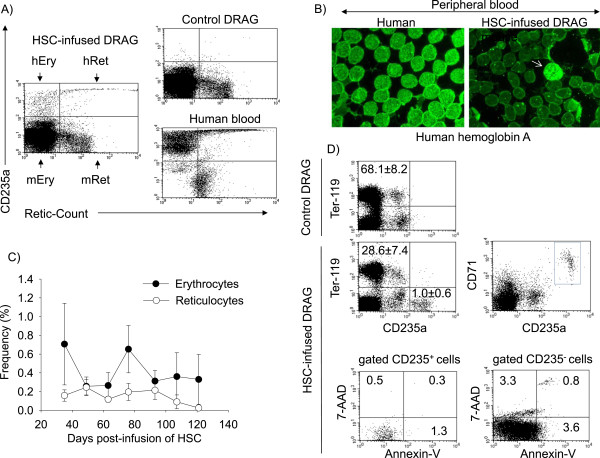
**DRAG mice infused with human HSC develop human RBCs. A)** Enumeration of human erythrocytes (hEry) and reticulocytes (hRet) in peripheral blood of DRAG mice by FACS using anti-human CD235a (glycophorin A) and Retic-Count (left panel). Controls are DRAG mice non-infused with HSCs (upper right panel) and human blood (lower right panel). **B)** Immunostaining of peripheral blood from HSC-infused DRAG mice (right) and human blood (left) with anti-human haemoglobin A. Arrow shows the presence of human RBCs. **C)** Frequency of human erythrocytes and reticulocytes in blood of HSC-infused DRAG mice (n = 10). Data represent mean ± SD of mice analysed individually. **D)** Bone marrow from HSC-infused (n = 3) and control (non-infused) (n = 7) DRAG mice was stained with anti-human CD235a and anti-mouse Ter-119 (upper left panels). Data represent mean ± SD of mice analysed individually. Staining with anti-human CD235a and anti-human CD71 showed that that the nucleated human RBC precursors expressed CD71 (upper right panel) (n = 1). Annexin-V and 7-AAD staining of human RBC precursors (CD235^+^) and non-erythroctyc precursors (CD235^−^) in the bone marrow of HSC-infused DRAG mice (lower panels) (n = 1).

RBC precursors from the bone marrow are released to the peripheral blood as reticulocytes (immature RBCs), which differentiate toward erythrocytes (mature RBCs) within 1–4 days of blood circulation [33]. Peripheral blood from HSC-infused DRAG mice was examined by FACS using anti-human CD235a (glycophorin A) and Retic-Count to estimate the frequency of human reticulocytes (CD235a^+^/Retic-Count^+^) and erythrocytes (CD235a^+^/Retic-Count^−^). Retic-Count is a solution of thiazole orange that binds to ribosomal and mitochondrial RNA present in reticulocytes but absent in erythrocytes [34]. As illustrated in Figure 3A, human reticulocytes and erythrocytes were present in blood of HSC-infused DRAG mice, but not in control (non-infused) DRAG mice. Interestingly, the pattern of CD235a expression in human erythrocytes and reticulocytes of DRAG mice was similar to that of human blood (Figure 3A, right lower panel) since reticulocytes expressed higher levels of CD235a than the erythrocytes. Human RBCs in the peripheral blood of HSC-infused DRAG mice were also visualized by immunohistochemistry using anti-human haemoglobin A (Figure 3B).

Human RBCs in the blood of DRAG mice were detected for up four-months after infusion of human HSCs, though the human haematocrit only represented 0.2-1% of the total haematocrit (Figure 3C). To determine whether the low human haematocrit in DRAG mice was due to insufficient differentiation of human HSC toward the erythrocytic lineage (erythropoiesis), the bone marrow was examined by FACS. As illustrated in Figure 3D (upper left panels), the frequency of human RBCs precursors (CD235a^+^) was significantly lower than the frequency of mouse RBC precursors (Ter-199^+^), despite a high frequency of human cells (CD45^+^, 45 ± 16%) and human HSC (CD45^+^CD34^+^, 4.7 ± 0.7%) in the bone marrow. The human RBC precursors in bone marrow expressed human CD71 (transferrin receptor) (Figure 3D, upper right panel), a marker expressed by nucleated human RBC precursors that is lost after enucleation and differentiation toward reticulocytes and erythrocytes [35].

To determine whether the human RBC precursors could have been eliminated by apoptosis, the bone marrow of HSC-infused DRAG mice was stained with Annexin V and 7-AAD (markers for early and late apoptosis) and human CD235. As illustrated in Figure 3D (lower panels) the human RBC precursors (CD235^+^) showed minimal apoptosis and comparable to that of non-erythrocytic (CD235^−^) cells, which argues against the human erythrocytic precursors being deleted by apoptosis. The results thus indicated that the low frequency of human RBCs in the blood of DRAG mice accounts for insufficient differentiation of human HSCs toward the erythrocytic lineage.

### DRAG mice challenged with *P. falciparum*sporozoites develop blood stage parasitaemia and are infectious to *Anopheles stephensi*mosquitoes

**Figure 4:**
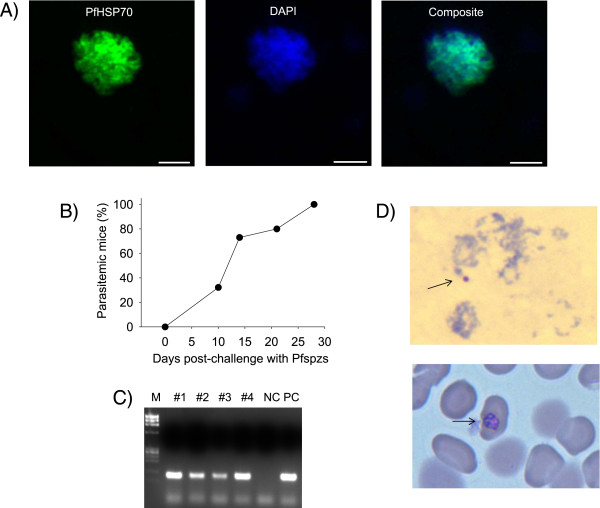
**DRAG mice sustain the complete vertebrate life cycle of** ***P. falciparum*** **infection. A)** HSC-infused DRAG mice were challenged i.v. with 10^5^ infectious *P. falciparum* sporozoites and five days later the livers were stained with anti-PfHSP70 (green) and DAPI (DNA, blue) and analysed by confocal microscopy. Data show a representative *P. falciparum* liver stage schizont. Scale bars are 10 μm. **B)** DRAG mice were challenged with sporozoites as above and followed for blood stage parasitaemia by PCR using primers specific for *Plasmodium* 18S rRNA. Data show percentage of parasitaemic mice (n = 31) from five independent challenges. **C)** PCR analysis in a group of four HSC-infused DRAG mice at day 28 post-challenge. NC, negative control; PC, positive control; M, DNA molecular markers. **D)** Giemsa staining of thick (upper panel) and thin (lower panel) blood smears from infected DRAG mice. Arrows show the presence of *P. falciparum* rings in thick smears and trophozoites in thin smears.

Since DRAG mice develop human hepatocytes, Kupffer cells, endothelial cells, and erythrocytes, it was next investigated whether they sustain the vertebrate life cycle of *P. falciparum*. For this, DRAG mice were challenged i.v. with *P. falciparum* sporozoites (NF54, 10^5^ per mouse) and five days later the livers were examined by immunohistochemistry using anti-*P. falciparum* HSP70 (PfHSP70). *Plasmodium falciparum* liver stage schizonts were detected in the livers (Figure 4A), which demonstrated that human hepatocytes developed by DRAG mice sustain infection by *P. falciparum* sporozoites.

To determine whether the liver stage parasites can transition toward blood stage parasites, additional groups of DRAG mice were challenged with *P. falciparum* sporozoites as above and they were follow-up for blood stage parasitaemia. As illustrated in Figure 4B and C, all DRAG mice challenged with NF54 *P. falciparum* sporozoites (a total of 31 mice from five independent challenges) developed blood stage parasitaemia as measured by PCR with a pre-patent period ranging between 10 to 28 days post-challenge. As measured by Giemsa-stained blood smears the levels of parasitaemia were low (3–5 parasites/μl of blood) (Figure 4D), which is explained by the low level of human haematocrit developed by DRAG mice (Figure 3C). DRAG mice (n = 4) challenged with sporozoites of *P. falciparum* strain 3D7 also became parasitaemic by day 17 post-challenge, which indicated that DRAG mice also sustain the vertebrate life cycle of 3D7 malaria parasites.

**Figure 5:**
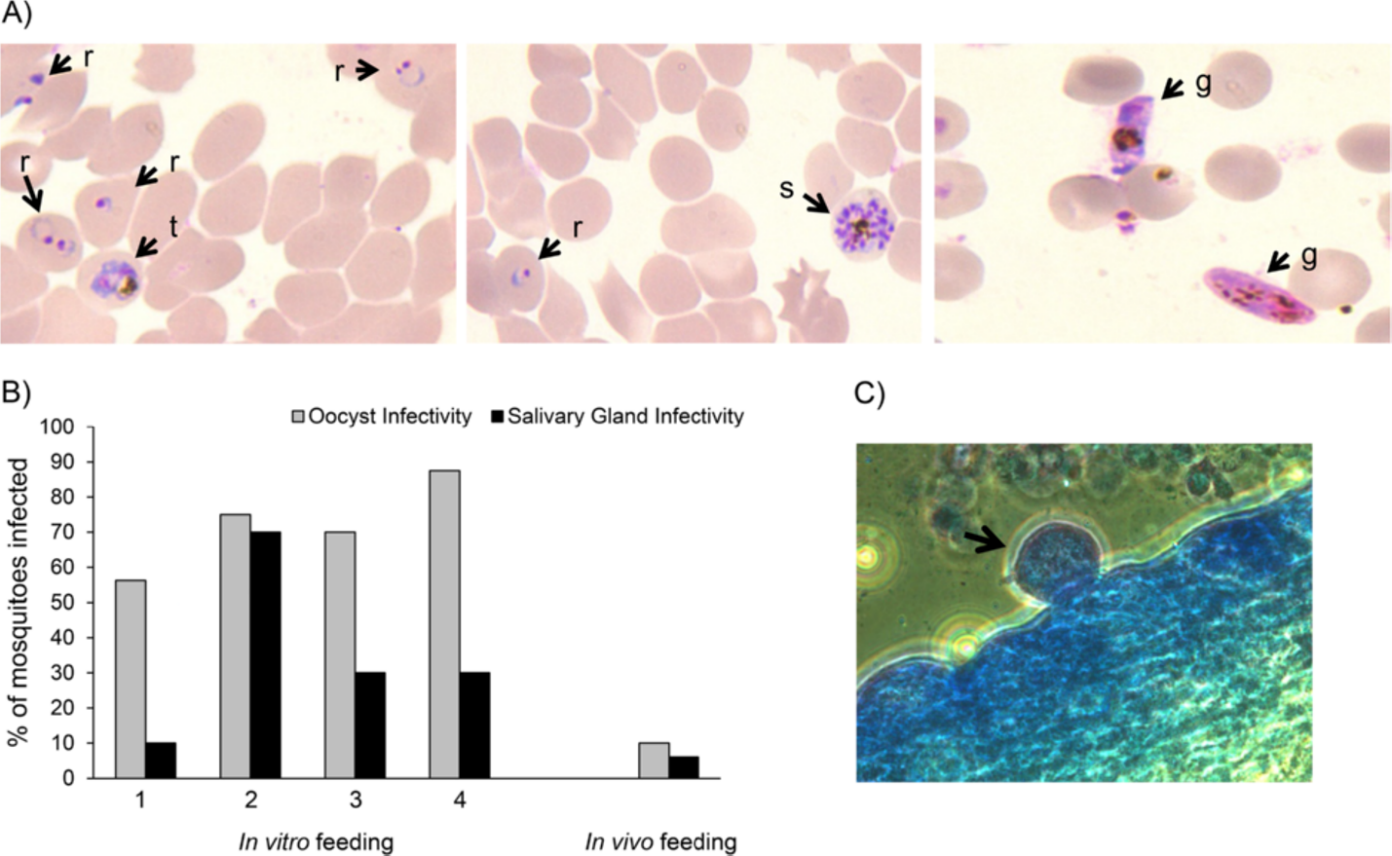
***Plasmodium falciparum*** **blood stage parasites from infected DRAG mice expand in** ***in vitro*** **cultures. A)** Giemsa staining of *in vitro* cultures showing presence of rings (r), trophozoites (t) and schizonts (s) (left and middle panels) and gametocytes (right panel). **B)** *Anopheles stephensi* mosquitoes were fed on the gametocyte cultures (four independent membrane feedings) or directly on an infected DRAG mouse (day 28 post-challenge). Oocysts in midgut and sporozoites in salivary glands were examined at days 7 and 14 post-feeding, respectively. Data represent frequency of infected mosquitoes among 25–35 mosquitoes analysed individually. **C)** Oocysts in midgut of mosquitoes fed on an infected DRAG mouse.

*Plasmodium falciparum* blood stage parasites from the peripheral blood of infected DRAG mice expanded upon *in vitro* culture. Figure 5A (left and middle panels) shows the presence of *P. falciparum* rings (r), trophozoites (t), and schizonts (s) in the *in vitro* cultures. The asexual blood stage parasites differentiated into gametocytes (g) (Figure 5A, right panel), and developed into oocysts and sporozoites in *An. stephensi* mosquitoes fed on the gametocyte cultures (Figure 5B). *Anopheles stephensi* mosquitoes directly fed on infected DRAG mice (day 28 post-challenge) also developed oocysts and sporozoites (Figure 5B and C). In aggregate these results indicated that DRAG mice sustain the complex vertebrate life cycle of *P. falciparum*.

### DRAG mice elicit immune responses to *P. falciparum*blood stage parasites and self-cure the infection

**Figure 6:**
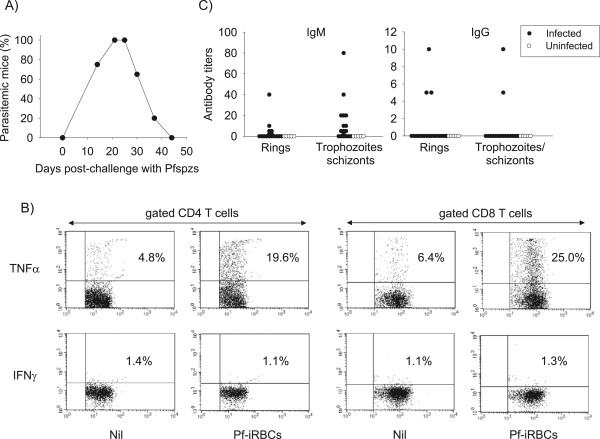
***Plasmodium falciparum*** **-infected DRAG mice elicit specific antibodies and self-cure blood stage infection. A)** DRAG mice (n = 18) were challenged with *P. falciparum* sporozoites as in Figure 4 and followed for blood stage parasitaemia by PCR and blood smears. All mice were infected by day 28 post-challenge but mice self-cured the infection by day 45 post-challenge. **B)** Spleen cells from infected DRAG mice (day 28 post-challenge) were stimulated *in vitro* with protein extracts of *P. falciparum* infected red blood cells (Pf-iRBCs) for 2 days or left unstimulated and analysed by FACS using anti-human CD3, CD4, CD8, TNF, and IFNγ. Numbers on the histograms represent frequencies of TNF and IFNγ positive cells among gated human CD4 and CD8 T cells from pooled mice (n = 3). **C)** Titers of human IgM (left panel) and IgG (right panel) antibodies to Pf-iRBCs using IFA slides coated with synchronized rings and trophozoites/schizonts. Data represent antibody titers in mice analysed individually at day 28 post-challenge. Fourteen out of 18 infected DRAG mice (closed circles) elicited specific antibodies, whereas uninfected DRAG mice (n = 5) did not have specific antibodies (open circles).

**Figure 7:**
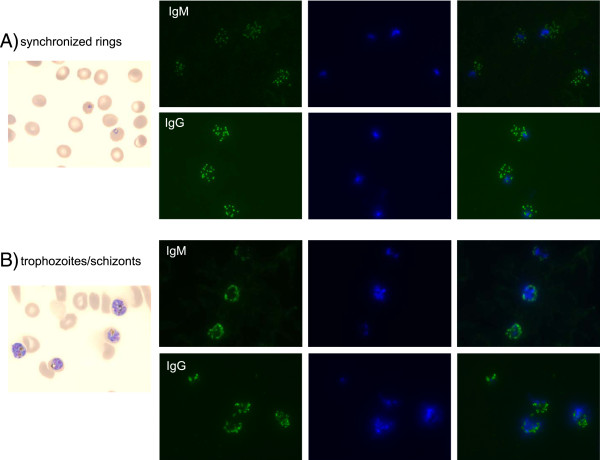
**Representative IFA staining pattern of** ***P. falciparum*** **antibodies elicited by DRAG mice.** Staining of *P. falciparum* synchronized rings **(A)** and trophozoites/schizonts **(B)** with sera from infected DRAG mice and revealed with anti-human IgM (green) or IgG (green) and counterstained with DAPI (DNA, blue). Giemsa staining of synchronized rings and trophozoites/schizonts in cultures is shown in left panels.

Longitudinal analysis in additional groups of DRAG mice (n = 18) revealed that all mice self-cured by day 45 post-challenge with sporozoites (Figure 6A). Since antibodies and cytotoxic cytokines play a role in the clearance of blood stage parasites [36, 37], and DRAG mice develop a functional human immune system [21] it was next investigated whether infected DRAG mice could have elicited cellular and antibody responses to *P. falciparum* blood stage parasites. Splenic human CD4 and CD8 T cells from infected DRAG mice (day 28 post-challenge, n = 3) that were stimulated *in vitro* with protein extracts of *P. falciparum* infected red blood cells (Pf-iRBCs) produced TNF, but not IFNγ as measured by FACS (Figure 6B). In addition, most DRAG mice (14 out of 18) examined at day 28 post-challenge elicited specific human IgM and IgG antibodies, as measured by IFA using slides coated with *P. falciparum* synchronized rings and trophozoites/schizonts (Figure 6C). The IFA staining pattern of antibodies to *P. falciparum* rings and trophozoites/schizonts was consistent among the individual DRAG mice (Figure 7). The results indicating that DRAG mice elicited specific cellular and antibody responses might thus account for their ability to clear *P. falciparum* blood stage infection.

## Discussion

This study provides evidence for the first humanized mouse model sustaining the complete life cycle of *P. falciparum* (strains NF54 and 3D7). Humanized DRAG mice infused with human HSCs developed human hepatocytes, Kupffer cells, liver endothelial cells, and erythrocytes and upon challenge with *P. falciparum* sporozoites sustained liver-to-blood stages of infection and supported transmission to *An. stephensi* mosquitoes. In the SCID/uPA and FRG mice it has only been demonstrated that the infused human hepatocytes can be infected with *P. falciparum* sporozoites that mature and can infect exogenously injected human RBCs [11]. Likewise, NSG mice have only demonstrated *P. falciparum* blood stage infection using mouse adapted parasite strains as long as mice were injected with human RBCs [16, 17].

Unlike other humanized mouse models such as NSG, DRAG mice express HLA class II molecules, and previous studies demonstrated that expression of HLA class-II molecules in humanized mice is critical for reconstitution of human T and B cells and immunoglobulin class switching [21, 38]. The ability of DRAG mice to develop human hepatocytes seems to be unrelated to HLA-II expression since prior studies in NSG mice (which do not express human HLA-II molecules) showed development of human hepatocytes from human HSCs. However there is controversy on whether transdifferentiation of human HSC into hepatocytes or cell fusion between human HSC and mouse hepatocytes accounts for the generation of human hepatocytes in humanized mice [39]. This study shows for the first time that that hepatocytes developed from HSCs sustain infection by *P. falciparum* sporozoites.

Despite the low numbers of human hepatocytes developed by DRAG mice, all DRAG mice challenged with sporozoites (n = 49) sustained liver stage infection, since they developed blood stage parasitaemia (Figures 4B and 6A). The high susceptibility of DRAG mice to liver stage infection might relate to their ability to develop human Kupffer cells and liver endothelial cells, which play a critical role for recruitment of sporozoites to the liver and infection of hepatocytes. Studies by Frevert’s group in rodent models indicated that sporozoite infection of the liver involves three steps: arrest in the sinusoid by interaction with endothelial cells, gliding to and passage through Kupffer cells, and invasion of hepatocytes [30]. Other authors however have found a minimal role of sporozoite traversal of Kupffer cells, but a role of sporozoite traversal of liver endothelial cells for effective infection of hepatocytes [31]. The need of accessory (Kupffer and/or endothelial) cells for effective sporozoite infection of hepatocytes has also became evident in *in vitro* studies, since cultures of isolated human hepatocytes (devoid of Kupffer and endothelial cells) have a very low rate of infection [40]. The ability of DRAG mice to reconstitute human Kupffer and liver endothelial cells may thus account for their high susceptibility to *P. falciparum* liver stage infection, despite the low numbers of human hepatocytes.

DRAG mice challenged with *P. falciparum* sporozoites developed blood stage parasitaemia and supported transmission to *An. stephensi* mosquitoes. The low level human haematocrit and hence parasitaemia in DRAG mice was a consequence of poor differentiation of human HSC toward the erythrocytic lineage. This was clear since bone marrow of DRAG mice contained low numbers of human erythrocytic precursors (CD45^−^CD235^+^) and the human erythrocytic precursors were not undergoing apoptosis. Previous studies in NSG mice (that unlike DRAG mice do not express human HLA II molecules) have led to controversial results on their ability to generate human RBCs [41, 42]. As found in this study, DRAG mice developed human erythrocytes and reticulocytes for several months upon the infusion of the human HSCs, albeit at low numbers. Approaches that increase the endogenous levels of human RBCs in DRAG mice are expected to increase the levels of parasitaemia and would add more value for this new *P. falciparum* mouse model.

DRAG mice sustained *P. falciparum* blood stage parasitaemia for a limited period of time since all mice self-cured by days 30–45 after the challenge with sporozoites. The ability of DRAG mice to clear the infection was associated with development of antibodies (human IgM and IgG) and TNF-mediated CD4 and CD8 T cell responses to the blood stage parasites. Numerous studies in humans and rodent malaria models indicated that high level parasitaemia induces immunosuppression by dendritic cell dysfunction and stimulation of regulatory T cells [13, 43, 44], and studies have shown that maintaining low level blood stage parasitaemia by chloroquine treatment allows development of protective immune responses to the blood stage parasites [13, 45]. Since DRAG mice develop a functional human immune system [21], the low level parasitaemia may thus account for the ability of DRAG mice to elicit specific immune responses and to clear parasites from the blood.

Considering that erythrocytes do not express HLA molecules [46] and infected erythrocytes cannot be directly eliminated by CD4 and CD8 T cells in a HLA-restricted manner, antibodies and factors secreted by immune cells are thought to be responsible for clearance of malaria parasites from the blood. Antibodies contribute to parasite clearance by several mechanisms such as (i) neutralization of proteins required for parasite invasion of erythrocytes, (ii) activation of complement cascade, (iii) Fc receptor mediated phagocytosis and (iv) Fc-receptor-mediated lysis by NK and NKT cells (ADCC, antibody-dependent cell cytotoxicity) [1]. Cellular immunity further contributes to clearance of blood stage parasites by secretion of cytokines, such as TNF, and toxic NO molecules [43, 44]. DRAG mice elicited both antibodies and TNF-mediated responses to *P. falciparum* blood stage parasites. The role of antibodies in clearing blood stage parasites in DRAG mice is further supported by the fact that human monoclonal antibodies generated using human B cells from infected DRAG mice showed strong anti-parasitic activity *in vitro* (Wijayalath et al., manuscript in preparation). However, some DRAG mice (4 out of 18 tested) did not have detectable specific antibodies as measured by IFA, which further suggests that cellular-mediated responses also contributed to clearance of *P. falciparum* parasites from the blood. The beneficial role of TNF in malaria has been demonstrated in animals treated with neutralizing TNF monoclonal antibodies, which prevented clearance of parasitaemia [47] *.* Though DRAG mice are deficient in mouse innate immunity (i.e., NK and dendritic cells) due to the IL2Rgc KO mutation [48, 49], DRAG mice develop mouse monocytes that could have also contributed to the clearance of *P. falciparum* blood stage parasites.

In aggregate, this study provides first evidence for a new human-immune-system mouse model sustaining the complete vertebrate life cycle of *P. falciparum.* The proven ability of DRAG mice to develop a functional human immune system and to elicit specific responses to *P. falciparum* parasites may aid to decipher immune correlates of protection and identification of protective malaria antigens.

## Conclusions

This study provides first evidence for a humanized mouse model that sustains the complex life cycle of *P. falciparum* malaria (liver-to-blood stages of infection and transmission to mosquito) without the need of exogenous administration of human hepatocytes/erythrocytes or *P. falciparum* parasite adaptation. This study also demonstrated that DRAG mice elicit human antibody and cellular responses to *P. falciparum* blood stage parasites which may further help for discovery of protective malaria antigens and to decipher immune correlates of protection. Experimental approaches that can successfully increase the levels of endogenously-produced human erythrocytes and parasitaemia in DRAG mice are expected to add more value to this new *P. falciparum* mouse model.
